# Single-agent belantamab mafodotin for relapsed/refractory multiple myeloma: analysis of the lyophilised presentation cohort from the pivotal DREAMM-2 study

**DOI:** 10.1038/s41408-020-00369-0

**Published:** 2020-10-23

**Authors:** Paul G. Richardson, Hans C. Lee, Al-Ola Abdallah, Adam D. Cohen, Prashant Kapoor, Peter M. Voorhees, Axel Hoos, Karrie Wang, January Baron, Trisha Piontek, Julie Byrne, Scott Richmond, Roxanne C. Jewell, Joanna Opalinska, Ira Gupta, Sagar Lonial

**Affiliations:** 1 Dana Farber Cancer Institute, Boston, MA USA; 2 MD Anderson Cancer Center, Houston, TX USA; 3 University of Kansas Cancer Center, Fairway, KS USA; 4 Abramson Cancer Center, University of Philadelphia, Philadelphia, PA USA; 5 Mayo Clinic, Rochester, MN USA; 6 Levine Cancer Institute, Atrium Health, Charlotte, NC USA; 7 GlaxoSmithKline, Philadelphia, PA USA; 8 GlaxoSmithKline, Rockville, MD USA; 9 GlaxoSmithKline, Research Triangle Park, NC USA; 10 Emory University, Winship Cancer Institute, Atlanta, GA USA

**Keywords:** Drug development, Haematological diseases

## Abstract

DREAMM-2 (NCT03525678) is an ongoing global, open-label, phase 2 study of single-agent belantamab mafodotin (belamaf; GSK2857916), a B-cell maturation antigen-targeting antibody-drug conjugate, in a frozen-liquid presentation in patients with relapsed/refractory multiple myeloma (RRMM). Alongside the main study, following identical inclusion/exclusion criteria, a separate patient cohort was enrolled to receive belamaf in a lyophilised presentation (3.4 mg/kg, every 3 weeks) until disease progression/unacceptable toxicity. Primary outcome was independent review committee-assessed overall response rate (ORR). Twenty-five patients were enrolled; 24 received ≥1 dose of belamaf. As of 31 January 2020, ORR was 52% (95% CI: 31.3–72.2); 24% of patients achieved very good partial response. Median duration of response was 9.0 months (2.8–not reached [NR]); median progression-free survival was 5.7 months (2.2–9.7); median overall survival was not reached (8.7 months–NR). Most common grade 3/4 adverse events were keratopathy (microcyst-like corneal epithelial changes, a pathological finding seen on eye examination [75%]), thrombocytopenia (21%), anaemia (17%), hypercalcaemia and hypophosphatemia (both 13%), neutropenia and blurred vision (both 8%). Pharmacokinetics supported comparability of frozen-liquid and lyophilised presentations. Single-agent belamaf in a lyophilised presentation (intended for future use) showed a deep and durable clinical response and acceptable safety profile in patients with heavily pre-treated RRMM.

## Introduction

Despite improved outcomes with currently available therapies, including proteasome inhibitors (PIs), immunomodulatory agents and anti-CD38 monoclonal antibodies (mAbs), multiple myeloma (MM) remains a challenging disease that is incurable for most patients^1–4^. The typical MM clinical course includes frequent relapses and development of refractory disease^5^. With each successive line of treatment, the duration of response (DoR) and progression-free survival (PFS) get shorter^5,6^. Patients refractory to anti-CD38 mAbs have a poor prognosis and limited treatment options, with newer agents used in combination (such as selinexor plus dexamethasone) resulting in an overall response rate (ORR) of 26% in patients refractory to at least one PI, one immunomodulatory agent and daratumumab^7^. Thus, there remains a need for novel targets and therapies in relapsed/refractory MM (RRMM).

B-cell maturation antigen (BCMA), a member of the tumour necrosis factor receptor family, is expressed on the surface of all normal plasma cells and late-stage B cells, as well as on all malignant cells in all patients with MM^8,9^. BCMA promotes the maturation and long-term survival of normal plasma cells and is also essential for proliferation and survival of malignant plasma cells in MM^9^. Belantamab mafodotin (belamaf; GSK2857916) is a first-in-class, BCMA-targeted antibody-drug conjugate (ADC) consisting of a humanised, afucosylated anti-BCMA mAb fused to the cytotoxic payload monomethyl auristatin F (MMAF) by a protease-resistant maleimidocaproyl linker^10^. Belamaf specifically binds to BCMA and eliminates myeloma cells by a multimodal mechanism, including delivering mafodotin to BCMA-expressing malignant cells thereby inhibiting microtubule polymerisation, and inducing immune-independent ADC-mediated apoptosis; immune-dependent enhancement of antibody-dependent cellular cytotoxicity and phagocytosis; and release of markers characteristic of immunogenic cell death—a form of regulated cell death involving the release of a series of damage-associated molecular patterns (such as calreticulin and high-mobility group box 1) leading to an adaptive immune response^10–13^.

In the first-in-human, phase 1 DREAMM-1 study (NCT02064387), single-agent belamaf administered as a frozen-liquid presentation induced clinically meaningful (ORR: 60%; 95% confidence interval [CI]: 42.1–76.1), deep (54% of patients with a very good partial response [VGPR] or better) and durable responses (PFS: 12 months, 95% CI: 3.1–not estimable; DoR: 14.3 months, 95% CI: 10.6–not estimable) with median duration of follow-up of 12.5 months in patients previously treated with alkylators, PIs and immunomodulatory agents and refractory to the last line of therapy^14,15^. In a sub-group of patients previously treated with anti-CD38 mAbs, and refractory to both a PI and an immunomodulatory agent, an ORR of 38.5% was reported in patients receiving 3.4 mg/kg single-agent belamaf every 3 weeks (Q3W)^15^.

The pivotal, randomised, phase 2, DREAMM-2 study (NCT03525678) was designed to further assess the efficacy and safety of single-agent belamaf in patients who are refractory to an immunomodulatory agent and a PI and refractory and/or intolerant to a CD38-targeted mAb. Results from the DREAMM-2 primary analysis, in which patients received either belamaf 2.5 or 3.4 mg/kg in a frozen-liquid presentation intravenously Q3W, have been previously reported^16,17^. After approximately 13 months of follow-up, an ORR of 32% (97.5% CI: 21.7–43.6) and 35% (97.5% CI: 24.8–47.0) for the 2.5 and 3.4-mg/kg cohorts, respectively, was demonstrated in this heavily pre-treated patient population. Responses were deep (VGPR or better) in 58% and 66% of responders in each cohort, respectively. At the time of data cut-off, median PFS was 2.8 (95% CI: 1.6–3.6) and 3.9 (95% CI: 2.0–5.8) months in the 2.5-mg/kg and 3.4-mg/kg cohorts, respectively. The median DoR estimate was 11.0 (95% CI: 4.2–NR) and 6.2 (95% CI: 4.8–NR) months; median OS estimate was 13.7 (95% CI: 9.9–NR) and 13.8 (95% CI: 10.0–NR) months in the 2.5 and 3.4-mg/kg groups, respectively.

A refrigerated lyophilised powder presentation of belamaf was developed to improve supply chain robustness by eliminating frozen shipments and storage, and is the presentation intended for future clinical use. In order to gain clinical experience with the lyophilised presentation of belamaf, an independent, exploratory cohort of patients was included in the DREAMM-2 study to receive this alternative presentation. Herein, we report the analysis for this cohort.

## Methods

### Study design and treatment

The DREAMM-2 full study design has been reported previously^16^. In brief, this phase 2, open-label, two-arm, global, multicentre study consisted of a screening/baseline period after which patients in the main study were randomised to receive intravenous belamaf in a frozen-liquid presentation (2.5 or 3.4 mg/kg Q3W). An independent cohort of patients was enrolled to receive belamaf in a lyophilised presentation (3.4 mg/kg Q3W, selected on the basis of the results from the DREAMM-1 study^15^). As per International Conference on Harmonisation Q5E (ICHQ5E) guidance^18^, the liquid and lyophilised drug products have been deemed comparable for the purpose of safety and efficacy as both are administered intravenously, have the same formulation, are essentially identical upon dilution for administration, and have been demonstrated to be analytically comparable through extensive biochemical and functional characterisation studies (including primary and higher-order structures, bioassay and binding assays), and stability testing. This new presentation was supplied as a refrigerated lyophilised powder to be reconstituted with water for injection prior to dilution in normal saline. It was administered intravenously over ≥30 min on Day 1 of each 3-week cycle, until disease progression or unacceptable toxicity. No systemic pre-medication was given unless deemed necessary by the investigator. Corticosteroid eye drops and preservative-free lubricant eye drops were used in both eyes to mitigate corneal events, a known toxic effect of MMAF^19^ and commonly reported in DREAMM-1. At the discretion of patient and investigator, cooling eye masks could be applied from the start of belamaf infusion for approximately 1 h, and up to 4 h, as tolerated. Dose modifications (delays or reductions) were permitted to manage adverse events (AEs), or for medical or surgical and logistical reasons unrelated to treatment. Criteria for dose modifications and patient withdrawal from the study are shown in the study protocol (Supplementary Material). Patients in the lyophilised cohort followed the same assessments and procedures as in the main DREAMM-2 study^16^.

The study was conducted in accordance with the Declaration of Helsinki and Good Clinical Practice guidelines following approval by ethics committees and institutional review boards at each study site. All patients provided written informed consent.

### Patient population

Inclusion/exclusion criteria were the same for patients in the lyophilised study cohort and the main DREAMM-2 study^16^.

#### Key inclusion criteria

To be eligible for inclusion, patients had to be 18 years or older with an Eastern Cooperative Oncology Group performance status of 0–2 and a histologically or cytologically confirmed diagnosis of MM according to the International Myeloma Working Group (IMWG) criteria^20^. They must have undergone stem cell transplant (>100 days before enrolment) or been considered transplant-ineligible; had disease progression after ≥3 prior lines of anti-myeloma treatment; were refractory to both an immunomodulatory agent and a PI, and refractory and/or intolerant to an anti-CD38 mAb; and meet the criteria for adequate organ system function. Patients with mild or moderate renal impairment and history of cytopenias (without active conditions) were eligible.

#### Key exclusion criteria

Patients were excluded if they had received prior allogeneic stem cell transplant, BCMA-targeted therapy, had corneal epithelial disease at screening (except mild punctate keratopathy) or any serious and/or unstable medical, psychiatric disorder or other condition that could interfere with the patient’s safety, ability to provide informed consent or compliance to the study procedures. Full inclusion and exclusion criteria are included in the Supplementary Material.

### Endpoints and assessments

Analysis of the lyophilised cohort was an exploratory objective of the main DREAMM-2 study. Key endpoints were ORR (defined as the percentage of patients with a partial response or better, according to IMWG criteria)^20^ assessed by independent review committee (IRC), clinical benefit rate (minimal response or better), time to response (TTR), time to best response, DoR, time to progression, PFS, OS and safety. Investigator-assessed ORR was also recorded and will be reported elsewhere. The safety profile of lyophilised belamaf was monitored with clinical and laboratory assessments, the reporting of AEs graded (with the exception of keratopathy) according to the Common Terminology Criteria for Adverse Events (2010, version 4.03; see Supplementary Material)^21^ and the rate of discontinuations and dose adjustments. Keratopathy (defined as microcyst-like epithelial changes [MECs] to the corneal epithelium observed by eye examination, with or without symptoms), thrombocytopenia and infusion-related reactions (IRRs) were monitored as AEs of special interest (AESI). Baseline and subsequent eye examinations were performed pre-dose Q3W by an ophthalmologist or optometrist (full details are provided in the Supplementary Material). Corneal examinations and best-corrected visual acuity assessments (BCVA) were combined and graded on the basis of a keratopathy and visual acuity scale.

### Pharmacokinetic analysis

The pharmacokinetic (PK) profile of belamaf was assessed by measurement of belamaf, total mAb (with and without the cytotoxic payload MMAF) and cysteine-maleimidocaproyl MMAF (cys-mcMMAF; the cytotoxic moiety released from belamaf) in plasma collected at Cycles 1 and 3 from all patients. The bioanalytical methods used to quantify concentrations of these analytes were selective, accurate and reproducible (data not shown). The assay methods for belamaf and total mAb measure both free and soluble BCMA-complexed molecules. Individual PK parameters were calculated using standard non-compartmental methods.

### Statistical methods

The full analysis population comprised all patients enrolled in the lyophilised cohort of DREAMM-2, regardless of treatment administration. All patients who received ≥1 dose of lyophilised belamaf were included in the safety population. The sample size for this cohort was chosen based on feasibility in order to gain clinical experience with the lyophilised presentation. The probability of observing a ≥20% ORR was retrospectively calculated, with the assumption made that the true ORR was 33%, there would be a 95% probability of observing ≥20% ORR with 25 patients. For the ORR, two-sided exact 95% CI were reported; 95% CI are reported for other data. PFS, DoR and TTR were analysed using the Kaplan–Meier method. Descriptive statistics were used for efficacy endpoints, pre-treatment characteristics, AEs and PK parameters. All efficacy endpoints were assessed by the IRC. This study was overseen by an independent data monitoring committee. Direct comparisons to the main study were not intended or made, due to the non-randomised nature of enrolment into the lyophilised cohort and the relatively small numbers of patients enrolled. Analyses were carried out using Statistical Analysis System software (version 9.4).

## Results

### Patient disposition and baseline characteristics

**Fig. 1:**
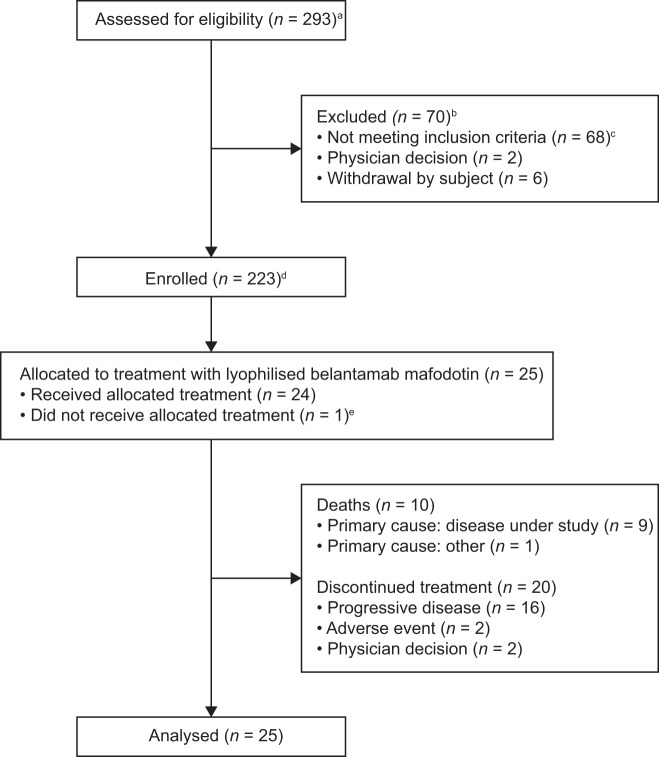
Patient disposition. **a** Between June 2018 and January 2019, 293 patients were screened for inclusion in the entire DREAMM-2 study. Between 5 December 2018 and 10 January 2019, 31 patients were screened for inclusion in the lyophilised presentation cohort. **b** Patients could have more than one reason for exclusion. **c** Five patients were excluded due to pre-existing corneal disease, as specified in the study protocol. **d** The remainder of enrolled patients were included in the main DREAMM-2 study previously reported^16^. Two patients in the main study were re-randomised and counted twice (once per each randomisation). **e** One patient was randomised to the belamaf 3.4 mg/kg lyophilised presentation, but actually received 3.4 mg/kg frozen-liquid presentation as first dose, and never received lyophilised presentation during the study.

Between 5 December 2018 and 10 January 2019, 31 patients were screened for the lyophilised presentation cohort at 9 sites in the USA and Australia. Twenty-five patients were allocated to treatment with the lyophilised presentation of belamaf (full analysis population) and 24 received the allocated treatment (safety population; 1 patient instead received frozen-liquid presentation; Fig. 1). At the data cut-off date (31 January 2020), patients had received a median of 3.5 treatment cycles (range: 1–17); median time on study treatment was 16.6 weeks (range: 3–60). Median duration of follow-up was 11.2 months (range: 1.8–14.5). At data cut-off, 17% (4/24) of patients were still receiving study treatment, and 83% (20/24) of patients had discontinued treatment (primary reason: progressive disease [67%]). Ten deaths were reported in this cohort: nine due to the disease under study, one had another cause.

Baseline characteristics are presented in Table 1. At screening, patients had received a median of 5 prior lines of therapy (range: 3–11). As per the inclusion criteria, all patients had received prior treatment with, and upon analysis were refractory to, a PI, an immunomodulatory agent, and an anti-CD38 mAb (daratumumab). Patients with high-risk cytogenetics (per IMWG criteria)^20^, international staging system stage III disease and extramedullary disease were well represented.

**Table 1:** Demographics, baseline disease, and clinical characteristics (full analysis population).

Characteristic	Lyophilised belantamab mafodotin 3.4 mg/kg (*N* = 25)
Age, median (range), years	68 (46–89)
18 to <65 years	10 (40)
65 to <75 years	9 (36)
≥75 years	6 (24)
Sex	
Male	14 (56)
Female	11 (44)
Race	
White or White European	21 (84)
Black or African American	3 (12)
Asian	1 (4)
Renal impairment per eGFR (mL/min/1.73 m^2^)	
Normal (≥90)	6 (24)
Mild (≥60 to <90)	13 (52)
Moderate (≥30 to <60)	6 (24)
Time from initial diagnosis, median (range), years	5.37 (1.92–10.28)
ISS disease stage at screening	
Stage I	7 (28)
Stage II	8 (32)
Stage III	10 (40)
Cytogenetic abnormalities	
*t*(11;14)	3 (12)
Del 13	6 (24)
Other^a^	9 (36)
High-risk cytogenetics^b^	7 (28)
17p13del	5 (20)
*t*(4;14)	1 (4)
*t*(14;16)	1 (4)
1q21+	5 (20)
Type of myeloma	
IgG	14 (56)
Non-IgG and missing	11 (44)
Light chain	
Kappa light chain	14 (56)
Lambda light chain	11 (44)
Extramedullary disease	6 (24)
Prior lines of therapy^c^	
Median (range)	5 (3–11)
≤4 lines	8 (32)
≥4 lines	17 (68)
Prior therapies received	
Proteasome inhibitor	25 (100)
Bortezomib	25 (100)
Carfilzomib	20 (80)
Ixazomib	6 (24)
Immunomodulatory agent	25 (100)
Lenalidomide	25 (100)
Pomalidomide	25 (100)
Thalidomide	4 (16)
Anti-CD38 monoclonal antibody	25 (100)
Daratumumab	25 (100)
Stem cell transplant	18 (72)
Refractory to prior therapies	
Proteasome inhibitor	25 (100)
Bortezomib	23 (92)
Carfilzomib	18 (72)
Ixazomib	5 (20)
Immunomodulatory agent	25 (100)
Lenalidomide	22 (88)
Pomalidomide	24 (96)
Thalidomide	3 (12)
Anti-CD38 monoclonal antibody	25 (100)
Daratumumab	25 (100)
Refractory to PI + immunomodulatory agent + anti-CD38 mAb^d^	25 (100)

*eGFR* estimated glomerular filtration rate, *IgG* immunoglobulin G, *ISS* International Staging System, *mAb* monoclonal antibody, *PI* proteasome inhibitor.

Data are *n* (%) unless otherwise specified.

^a^Other includes non-high risk, missing and not done.

^b^High-risk cytogenetics defined as having any of the following cytogenetic features: *t*(4;14), *t*(14;16), 17p13del or 1q21+.

^c^The number of prior lines of therapy is derived as the number of prior anti-cancer regimens received by a patient as reported on the electronic case report form. Combination therapy containing multiple components was counted as one regimen.

^d^All patients were refractory to a PI, an immunomodulatory agent, and refractory and/or intolerant an anti-CD38 mAb as per eligibility criteria. Refractory was defined as disease that is non-responsive while on primary or salvage therapy or progressing ≤60 days of last therapy.

### Efficacy

**Fig. 2:**
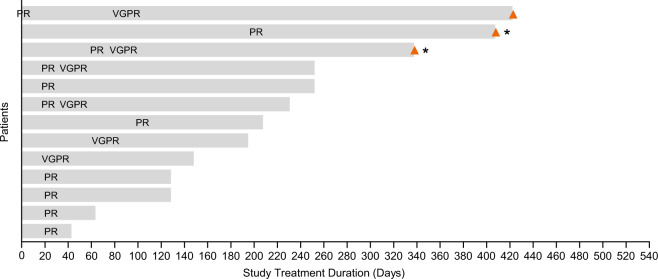
Time from randomisation to best confirmed response in responders (*n* = 13). Abbreviations: PR partial response, VGPR very good partial response. Responses were assessed by an independent review committee according to International Myeloma Working Group criteria^20^. Orange triangles represent patients with study treatment ongoing. Asterisks represent patients with follow-up ongoing. Responses are indicated at the time of the first report of ≥PR, followed by best response, unless the two occurred concurrently.

**Fig. 3:**
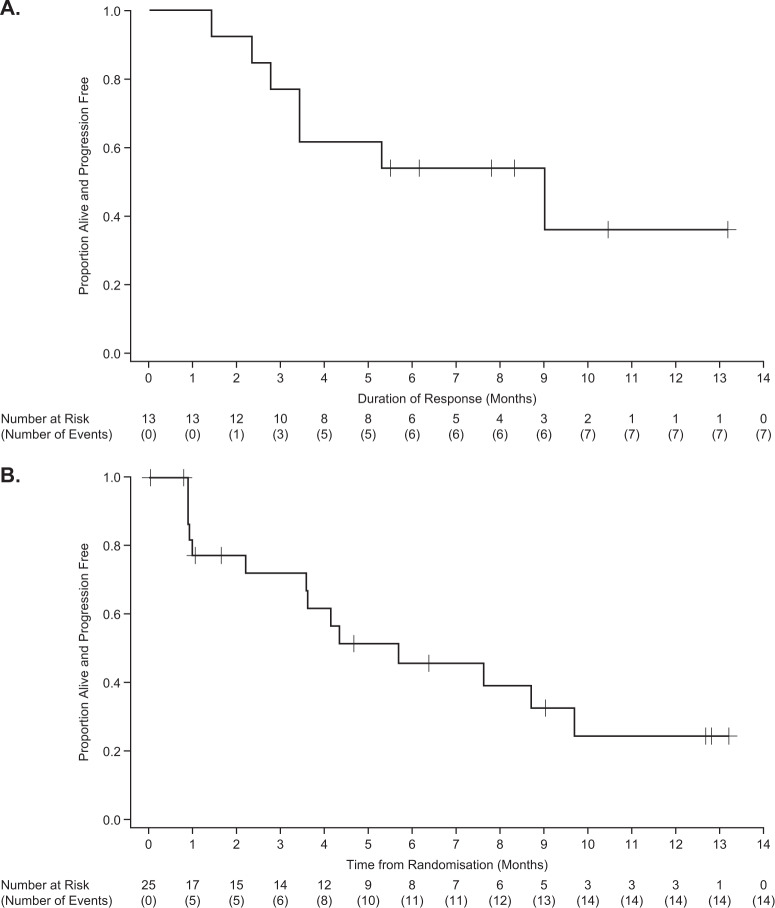
Duration of response (A) and progression-free survival (B) full analysis population. Responses were assessed by an independent review committee according to International Myeloma Working Group criteria^20^.

The IRC-assessed ORR was 52% (95% CI: 31.3–72.2). A VGPR was seen in 24% (6/25) of patients (46% [6/13] of responders) (Fig. 2 and Table 2). The IRC-assessed clinical benefit rate (minimal response or better) was 56% (95% CI: 34.9–75.6). The median (95% CI) TTR was 0.9 months (0.8–2.3) and the median time to best response was 1.4 months (0.8–2.9). At the time of data analysis, the median DoR was 9.0 months (95% CI: 2.8–NR) (Fig. 3A). Based on the Kaplan–Meier curve, the probability of maintaining a response ≥6 months was estimated to be 54% (95% CI: 25–76). The median PFS was 5.7 months (95% CI: 2.2–9.7 [Fig. 3B]). At data cut-off, median OS was not reached (95% CI: 8.7 months–NR).

**Table 2:** Independent review committee-assessed response (full analysis population).

Response category	Lyophilised belantamab mafodotin 3.4 mg/kg (*N* = 25)
Best response	
Very good partial response (VGPR)	6 (24)
Partial response (PR)	7 (28)
Minimal response (MR)	1 (4)
Stable disease	4 (16)
Progressive disease	6 (24)
Not evaluable	1 (4)
Overall response rate (ORR)^a^	13 (52) (95% CI: 31.3–72.2)
Clinical benefit rate (CBR)^b^	14 (56) (95% CI: 34.9–75.6)

Data are *n* (%) unless otherwise specified. No patients had stringent complete response (sCR) or complete response (CR).

^a^ORR included sCR+CR + VGPR + PR.

^b^CBR included sCR+CR + VGPR + PR + MR.

### Safety

Overall, 100% (24/24) of patients experienced ≥1 AE. The most common AEs (any grade) were keratopathy (MECs, changes to the corneal epithelium observed by eye examination with or without symptoms), thrombocytopenia, fatigue, blurred vision, dry eye, anaemia and back pain (Table 3). The most common grade 3/4 AEs were keratopathy (MECs), thrombocytopenia, anaemia, hypercalcaemia, hypophosphatemia, neutropenia and blurred vision (Table 3). Serious AEs (SAEs) were reported in 63% of patients (Supplementary Table 1) and were considered treatment related in 17% of patients. There was one death due to an SAE (due to cardiac failure; unrelated to study treatment).

**Table 3:** Most common AEs of any grade (occurring in ≥15% or an AE of special interest [AESI]) or grade 3/4 (occurring in ≥5%, safety population)^a^.

Event	Lyophilised belantamab mafodotin 3.4 mg/kg (*n* = 24)	
	Number of patients (%)	
	Any grade	Grade 3/4
Keratopathy (MECs)^b^	23 (96)	18 (75)
Thrombocytopenia^c^	11 (46)	5 (21)
Fatigue	11 (46)	0
Blurred vision^d^	9 (38)	2 (8)
Anaemia	6 (25)	4 (17)
Dry eye^e^	6 (25)	0
Back pain	6 (25)	1 (4)
Hyponatraemia	5 (21)	1 (4)
Intraocular pressure increased	5 (21)	0
Headache	5 (21)	1 (4)
Aspartate aminotransferase increased	5 (21)	0
Decreased appetite	5 (21)	0
Hypercalcaemia	4 (17)	3 (13)
Blood lactate dehydrogenase increased	4 (17)	0
Pyrexia^f^	4 (17)	1 (4)
Upper respiratory tract infection	4 (17)	0
Infusion-related reactions^f^	4 (17)	0
Hypophosphataemia	3 (13)	3 (13)
Neutropenia^g^	2 (8)	2 (8)

*AE* adverse event, *AESI* adverse event of special interest, *BCVA* best-corrected visual acuity, *KVA* keratopathy and visual acuity, *MECs* microcyst-like epithelial change.

Listed in order of decreasing frequency of any grade events.

^a^Events graded using the Common Terminology Criteria for Adverse Events criteria v4.03, with the exception of keratopathy (MECs)^21^.

^b^Corneal epithelium changes (an AESI) were observed on eye examination with or without changes in BCVA from baseline or symptoms. Graded per KVA scale.

^c^Thrombocytopenia (an AESI) includes preferred terms thrombocytopenia, haematoma and platelet count decreased.

^d^Blurred vision includes preferred terms vision blurred, diplopia and visual acuity reduced.

^e^Dry eye includes preferred terms dry eye and eye pruritus.

^f^Infusion-related reactions (an AESI) includes preferred terms infusion-related reaction, pyrexia, transfusion reaction and chills occurring ≤24 h of infusion.

^g^Neutropenia includes neutropenia and neutrophil count decreased.

Median dose intensity was 2.32 mg/kg Q3W (range: 1.0–3.4), which was lower than intended due to the incidence of dose reductions and delays. Dose reductions and delays occurred in 58% (14/24) and 71% (17/24) of patients, respectively. Of those with dose reductions, 71% (10/14) of patients had a single dose reduction to 2.5 mg/kg and 29% (4/14) had a second reduction to 1.92 mg/kg. In patients with dose delays, 59% (10/17) of patients had a single dose delay, 12% (2/17) had two dose delays and 29% (5/17) of patients had ≥3 dose delays. The median duration of dose delays was 21 days (range: 4–168). AEs leading to dose reductions (58%) and delays (79%) were common; 2 patients (8%) permanently discontinued treatment due to AEs (keratopathy [MECs] in 1 patient, cardiac failure in 1 patient). Permanent treatment discontinuation due to AEs was considered treatment related in 1 patient (4%). The most common AEs leading to dose reductions (occurring in ≥5% of patients) included keratopathy (MECs; in 46% of patients), thrombocytopenia (8%) and blurred vision (8%); patients could have more than one AE leading to dose reduction. Keratopathy (MECs; 75%) and blurred vision (25%) were the most common AEs leading to dose delays.

Thrombocytopenia (which included thrombocytopenia, haematoma and platelet count decreased) was reported in 46% of patients, with 21% of patients experiencing grade 3/4 events (Table 3). IRRs (including terms IRR, pyrexia, transfusion reaction and chills occurring ≤24 h of infusion) occurred in 17% (4/24) of patients, with no grade 3/4 events. In patients with IRRs, the first occurrence was typically with first infusion (in 75% [3/4] patients); 2/4 patients experienced a single IRR and 2/4 had two IRRs; IRRs resolved in all patients. Although not protocol mandated, 46% of patients received at least one prophylactic pre-medication for IRRs, with 29% of patients receiving prophylactic pre-medication at Cycle 1. In terms of drug class, 33% of patients in the safety population received an analgesic (paracetamol), 42% received an antihistamine and 25% received a steroid as prophylactic pre-medication for IRRs.

Keratopathy (MECs) was the most frequent AE (96%); grade 1/2 (mild/moderate) events were recorded in 21% (5/24) patients and grade 3 (severe) events in 75% (18/24) patients. No grade 4 events occurred. In patients with grade ≥2 events (*n* = 21), the median time to onset of the first occurrence of keratopathy (MECs) was 23 days (range: 18–283). The onset of keratopathy (MECs) was reported in 58% of patients in the safety population at Cycle 1, in 83% of patients by Cycle 2, in 92% of patients by Cycle 4 and reached the maximum reported incidence of 96% after Cycle 10. At data cut-off, 52% (11/21) of patients with ≥grade 2 keratopathy (MECs) recovered from the first occurrence, with a median duration of first occurrence of 127 days (range: 23–278). Among the 11 patients who recovered, 5 recovered after treatment discontinuation, 5 recovered with dose delay or dose reduction, and 1 recovered without dose modification. At data cut-off, the first occurrence of ≥grade 2 keratopathy (MECs) had not resolved in the remaining 48% (10/21) of patients. Of these, 20% (2/10) of patients were on treatment, 50% (5/10) were no longer in follow up due to death or loss to follow-up and 30% (3/10) were still in follow-up. Keratopathy (MECs) was the most common AE leading to dose delays (75%) and reductions (46%). Dose delays due to keratopathy (MECs) began at Week 4, while dose reductions began later, at Week 7.

BCVA declined to 20/50 or worse in the better seeing eye at least once during or after the treatment period in 33% (8/24) of patients. Median time to onset for the first occurrence was 57 days (range: 39–146). As of the last follow-up, 100% (8/8) patients had recovered (BCVA better than 20/50 in the better seeing eye) with median time to recovery of 21.5 days (range: 16–43). Two patients had a transient worsening of their vision (BCVA worse than or equal to 20/200) in one eye only; however, both patients saw an improvement in BCVA (i.e., returned to baseline during follow-up). In 1 patient, the event occurred 61 days after the last dose (treatment discontinued due to progressive disease) and had resolved 21 days later; in the other patient, the event occurred after the first dose but was resolved prior to administration of the second dose. This patient has remained on treatment without a further occurrence; follow-up is ongoing. No patients had a transient worsening of vision to 20/200 in their better seeing eye.

Among patients with keratopathy (MECs), 83% reported symptoms (including blurred vision or subjective dry eye) and/or had a decrease in BCVA (2 or more lines decline in the better seeing eye). Overall, blurred vision and dry eye were the most common patient-reported corneal symptoms (38 and 25%, respectively), and were generally <grade 3 (Table 3). Median time to first occurrence of blurred vision and dry eye was 26 days (range: 19–247) and 45 days (range: 2–66), respectively. Median duration of first occurrence was 43 days (range: 26–178) and 115 days (range: 4–173), respectively. As of last follow-up, blurred vision had resolved in 56% (5/9) of affected patients, and dry eye resolved in 33% (2/6) of patients.

### Pharmacokinetics

Exposure measures (AUC and *C*_max_) were generally similar for the three analytes (belamaf, total mAb and cys-mcMMAF) after administration of either the frozen-liquid or lyophilised presentations of belamaf (Table 4). In population PK analyses, presentation was not a significant factor for belamaf PK (data not shown). After accounting for key covariates, PK behaviour of the three analytes was similar after administration of the frozen-liquid and lyophilised presentations.

**Table 4:** Summary of belantamab mafodotin, total monoclonal antibody and cys-mcMMAF pharmacokinetic parameter values at cycle 1 in patients receiving frozen-liquid^a^ or lyophilised presentation of belantamab mafodotin (safety population).

Parameter	2.5 mg/kg frozen-liquid (*n* = 95)		3.4 mg/kg frozen-liquid (*n* = 99)		3.4 mg/kg lyophilised (*n* = 24)	
	*n*	Value	*n*	Value	*n*	Value
Belantamab mafodotin						
AUC(0–τ) (μg•h/mL)	30	4666 (46)	20	5678 (40)	22	5946 (37)
* C*_max_ (μg/mL)	32	42.5 (26)	21	52.0 (20)	22	51.3 (18)
* t*_max_ (h)	32	0.78 (0.42–2.50)	21	0.70 (0.43–2.15)	22	0.75 (0.48–2.88)
* C*_trough_ (μg/mL)	69	2.43 (52)	71	2.54 (88)	20	3.41 (76)
Total monoclonal antibody						
AUC(0–τ) (μg•h/mL)	29	7305 (42)	18	9566 (42)	19	9029 (40)
* C*_max_ (μg/mL)	30	48.9 (30)	19	61.1 (27)	20	60.1 (18)
* t*_max_ (h)	30	1.75 (0.42–2.50)	19	1.87 (0.50–24.50)	20	0.65 (0.48–2.17)
* C*_trough_ (μg/mL)	66	5.27 (83)	71	5.98 (87)	18	8.13 (101)
Cys-mcMMAF						
AUC(0–168) (ng•h/mL)	14	84.3 (59)	12	109.4 (55)	7	81.6 (58)
* C*_max_ (pg/mL)	27	903 (64)	20	1148 (65)	19	1017 (61)
* t*_max_ (h)	27	22.83 (1.92–65.63)	20	23.84 (17.38–72.65)	19	24.08 (0.97–69.47)
* C*_trough_ (pg/mL)	82	NQ (NQ–58.0)	83	NQ (NQ–452.5)	24	NQ (NQ–NQ)

*AUC* area under the curve, *C*_*max*_ maximum observed plasma concentration, *C*_*trough*_ plasma concentration prior to next dose, *cys-mcMMAF* cysteine-maleimidocaproyl monomethyl auristatin F, *NQ* not quantifiable, *t*_*max*_ time of *C*_max_.

Data presented as geometric mean (%CVb), except *t*_max_ and *C*_trough_ for cys-mcMMAF, presented as median (minimum–maximum).

^a^Study population details, efficacy and safety analyses were previously reported^16^.

## Discussion

In this exploratory cohort of patients with heavily pre-treated RRMM, single-agent belamaf (3.4 mg/kg Q3W) in a lyophilised presentation demonstrated deep and durable anti-myeloma activity, with an ORR of 52%. Responses were deep, with 46% (6/13) of responders achieving a VGPR. ORRs were similar to those in patients with RRMM who were refractory to a PI and an immunomodulatory agent and exposed to anti-CD38 mAbs receiving single-agent belamaf 3.4 mg/kg Q3W in both the first-in-human DREAMM-1 study (ORR: 38.5% in this sub-group of 13 patients) and the previously published main DREAMM-2 study (ORR: 35% at 13-month follow-up). The ORR reported in this study compares favourably with STORM, the only other clinical trial designed to prospectively evaluate an anti-myeloma treatment (combination selinexor plus dexamethasone) in patients refractory to at least one PI, one immunomodulatory agent, and daratumumab (as in DREAMM-2), in which an ORR of 26% was reported^7^. The STORM study recruited patients previously exposed to bortezomib, carfilzomib, lenalidomide, pomalidomide, daratumumab and an alkylating agent, a similar population to this DREAMM-2 cohort in which all patients were exposed to bortezomib, lenalidomide, pomalidomide and daratumumab, and 80% of patients were exposed to carfilzomib. The median DoR in this study was 9.0 months (95% CI: 2.8–NR) after median follow-up of approximately 11 months; a median DoR of 4.4 months was reported in STORM^7^, suggesting that clinical responses to belamaf are durable, as was the case in the DREAMM-1 study^15^. The median PFS in this patient cohort was 5.7 months, while median OS was not reached (95% CI: 8.7 months–NR) even at this later time point.

As in the main DREAMM-2 study, belamaf had an acceptable safety profile, with no new safety concerns identified with the lyophilised presentation^16^. Based on previous clinical experience with belamaf and literature reports of MMAF-containing ADCs, thrombocytopenia was an AESI^19^. In this study, while common, thrombocytopenia was considered self-limiting and did not lead to treatment discontinuation. IRRs, as expected for biological agents including belamaf, were common, but resolved in all patients.

As expected, keratopathy (MECs) on eye examination was common, but events were generally limited to the epithelium (the superficial layer of the cornea) and rarely led to treatment discontinuation. Dry eye and blurred vision events were also common, but as with keratopathy (MECs), were effectively managed with dose delays and/or reductions and concomitant use of preservative-free lubricant eye drops. Corneal events associated with belamaf may be adequately managed by close liaison with eye care professionals and dose modifications (both delays and reductions), as clinically warranted. For patients with grade 1 events, treatment should be continued at the current dose (on the basis of the 2.5-mg/kg results from the main study)^17^. For grade 2 events, dosing should be withheld until corneal exam findings and changes in BCVA improve to a grade 1 event or better, when dosing should resume at the current dose. For grade 3 events, treatment should be withheld until corneal exam findings and changes in BCVA improve to grade 1 or better, when dosing should resume at a reduced dose of 1.9 mg/kg. Treatment should be permanently discontinued for grade 4 events.

The belamaf frozen-liquid presentation was primarily used in DREAMM-1 and in the main cohort of the pivotal DREAMM-2 study to evaluate safety and efficacy^14–16^. The refrigerated lyophilised presentation is intended for future clinical use, has been demonstrated to be analytically comparable to the liquid presentation, and is a more robust presentation since it eliminates the frozen shipment and storage requirements. From the patient perspective, either presentation of the drug product is essentially identical upon dilution for intravenous administration. After correction for covariates, no significant difference in PK behaviour was observed for the two presentations, and presentation was not a significant factor in the population PK and exposure–response analyses for belamaf. Belamaf is the first anti-BCMA agent with a multimodal mechanism of action, convenient dosing schedule and no requirement for combination with dexamethasone, making it potentially attractive for use in the real-world setting^22^. The data presented here, in combination with previously published data from DREAMM-1 and DREAMM-2, support single-agent lyophilised belamaf as a practical and effective treatment option for patients with heavily pre-treated RRMM.

## Supplementary information

Suppl Materials

Reporting Checklist

CONSORT Checklist

## Data Availability

Anonymised individual patient data and study documents can be requested from www.clinicalstudydatarequest.com.
