# Supplementary material for: Single-agent belantamab mafodotin for relapsed/refractory multiple myeloma: analysis of the lyophilised presentation cohort from the pivotal DREAMM-2 study
Source: Blood Cancer J. 2020 Oct 23;10(10):106. doi: 10.1038/s41408-020-00369-0 (PMC7584571; doi:10.1038/s41408-020-00369-0)
Supplement: Supplementary file 1 — Suppl Materials [file 41408_2020_369_MOESM1_ESM.pdf]

CONTENTS

INCLUSION/EXCLUSION CRITERIA 2
Inclusion Criteria 2
Exclusion Criteria 3
BELANTAMAB MAFODOTIN TREATMENT-RELATED CORNEAL EVENTS 5
Table S1. Serious Adverse Events and Adverse Events of Special Interest (Safety Population)[a] 6
Table S2. List of Sites, Study Investigators and Patients Recruited at Each Site 8
REFERENCES 9

INCLUSION/EXCLUSION CRITERIA
Inclusion Criteria

- Participants were eligible to be included in the study only if all the following criteria applied:
    - Provide signed written informed consent, which includes compliance with the requirements and restrictions listed in the consent form
    - Male or female, 18 years or older (at the time consent is obtained)
    - Eastern Cooperative Oncology Group (ECOG) performance status of 0–2
    - Histologically or cytologically confirmed diagnosis of multiple myeloma (MM) as defined according to international myeloma working group (IMWG) criteria[1], AND:
        - Has undergone stem cell transplant or is considered transplant ineligible, AND
        - Has failed at least three prior lines of anti-myeloma treatments, including an anti-CD38 antibody (e.g. daratumumab) alone or in combination, and is refractory to an immunomodulatory drug (i.e. lenalidomide or pomalidomide), and to a proteasome inhibitor (e.g. bortezomib, ixazomib or carfilzomib). The number of prior lines of therapy will be determined according to the guidelines in Rajkumar *et al*. 2015.[1]
    - Has measurable disease with at least one of the following:
        - Serum M-protein ≥0.5 g/dL (≥5 g/L)
        - Urine M-protein ≥200 mg/24h
        - Serum free light chain assay: Involved free light chain level ≥10 mg/dL (≥100 mg/L) and an abnormal serum free light chain ratio (<0.26 or >1.65)
    - Participants with a history of autologous stem cell transplant are eligible for study participation provided the following eligibility criteria are met:
        - Transplant was >100 days prior to study enrolment
        - No active infection(s)
        - Participant meets the remainder of the eligibility criteria
- Adequate organ system function, defined by:
    - Absolute neutrophil count ≥1.0 X 10^9/L; haemoglobin ≥8.0 g/dL; platelets ≥50 X 10^9/L; total bilirubin ≤1.5 X upper limit of normal (ULN) (isolated bilirubin ≥1.5 x ULN is acceptable if bilirubin is fractionated and direct bilirubin <35%); alanine aminotransferase ≤2.5 X ULN; estimated glomerular filtration rate (eGFR) (as calculated by Modified Diet in Renal Disease [MDRD] equation) ≥30 mL/min/1.73 m^2 ; spot urine (albumin/creatinine ratios) <500 mg/g (56 mg/mmol); left ventricular ejection fraction (echocardiogram) ≥45%.
- Female participants: Contraceptive use by men or women should be consistent with local regulations regarding the methods of contraception for those participating in clinical studies. A female participant is eligible to participate if she is not pregnant or breastfeeding, and at least one of the following conditions applies:
    - Is not a woman of childbearing potential (WOCBP)
      OR
    - Is a WOCBP and using a contraceptive method that is highly effective (with a failure rate of <1% per year), preferably with low user dependency, during the intervention period and for at least 80 days after the last dose of study intervention and agrees not to donate eggs (ova, oocytes) for the purpose of reproduction during this period. The investigator should evaluate the effectiveness of the contraceptive method in relationship to the first dose of study intervention.
        - A WOCBP must have a negative highly sensitive serum pregnancy test (as required by local regulations) within 72 hours before the first dose of study intervention.

    - The investigator is responsible for review of medical history, menstrual history, and recent sexual activity to decrease the risk for inclusion of a woman with an early undetected pregnancy.
- Male Participants: Contraceptive use by men or women should be consistent with local regulations regarding the methods of contraception for those participating in clinical studies. Male participants are eligible to participate if they agree to the following during the intervention period and for at least 140 days:
  - Refrain from donating sperm

  PLUS either:

  - Be abstinent from heterosexual intercourse as their preferred and usual lifestyle (abstinent on a long-term and persistent basis) and agree to remain abstinent.

    OR

  - Must agree to use contraception/barrier as detailed below:
    - Agree to use a male condom and female partner to use an additional highly effective contraceptive method with a failure rate of <1% per year as when having sexual intercourse with a woman of childbearing potential who is not currently pregnant.
- All prior treatment-related toxicities (defined by National Cancer Institute- Common Toxicity Criteria for Adverse Events (NCI-CTCAE), version 4.03,[2] must be ≤Grade 1 at the time of enrolment except for alopecia and Grade 2 peripheral neuropathy.
- (France only) A participant will be eligible for inclusion in this study only if either affiliated to or a beneficiary of a social security category.

Exclusion Criteria

- Participants satisfying any of these criteria were not eligible for assignment to treatment:

  - Systemic anti-myeloma therapy within ≤14 days or 5 half-lives, whichever is shorter, or plasmapheresis within 7 days prior to the first dose of study drug
  - Systemic treatment with high dose steroids (equivalent to ≥60 mg prednisone daily for ≥4 days) within the past 14 days if administered to treat MM or non-MM disease
  - Symptomatic amyloidosis, active ‘polyneuropathy, organomegaly, endocrinopathy, myeloma protein, and skin changes’ (POEMS) syndrome, active plasma cell leukemia at the time of screening
  - Prior allogeneic stem cell transplant
  - Current corneal epithelial disease except mild punctate keratopathy
  - Use of an investigational drug within 14 days or 5 half-lives, whichever is shorter, preceding the first dose of study drug. Prior treatment with a monoclonal antibody within 30 days of receiving the first dose of study drugs
  - Prior B-cell Maturation Antigen (BCMA) targeted therapy
  - Evidence of active mucosal or internal bleeding
  - Any major surgery within the last four weeks
  - Presence of active renal condition (infection, requirement for dialysis or any other condition that could affect participant’s safety). Participants with isolated proteinuria resulting from MM are eligible, provided they have adequate organ system function, defined as:
    - Absolute neutrophil count ≥1.0 X 10^9/L; haemoglobin ≥8.0 g/dL; platelets ≥50 X 10^9/L; total bilirubin ≤1.5 X ULN (isolated bilirubin ≥1.5 x ULN is acceptable if bilirubin is fractionated and direct bilirubin <35%); alanine aminotransferase ≤2.5 X ULN; eGFR (as calculated by MDRD equation) ≥30

mL/min/ 1.73 m^2; spot urine (albumin/creatinine ratios) <500 mg/g (56 mg/mmol); left ventricular ejection fraction (echocardiogram) ≥45%.

- Any serious and/or unstable pre-existing medical, psychiatric disorder or other conditions (including lab abnormalities) that could interfere with participant's safety, obtaining informed consent or compliance to the study procedures.
- Current unstable liver or biliary disease per investigator assessment defined by the presence of ascites, encephalopathy, coagulopathy, hypoalbuminemia, oesophageal or gastric varices, persistent jaundice, or cirrhosis.
    - Note: Stable chronic liver disease (including Gilbert's syndrome or asymptomatic gallstones) or hepatobiliary involvement of malignancy is acceptable if participant otherwise meets entry criteria.
- Malignancies other than disease under study are excluded, except for any other malignancy from which the participant has been disease-free for more than years and, in the opinion of the principal investigators and GSK Medical Monitor, will not affect the evaluation of the effects of this clinical trial treatment on the currently targeted malignancy. Participants with curatively treated non-melanoma skin cancer may be enrolled.
- Evidence of cardiovascular risk including any of the following:
    - QTcF interval QTcF > 480 msec (the QT interval values must be corrected for heart rate by Fridericia's formula [QTcF]
    - Evidence of current clinically significant uncontrolled arrhythmias, including clinically significant electrocardiogram abnormalities such as 2nd degree (Type II) or 3rd degree atrioventricular (AV) block.
    - History of myocardial infarction, acute coronary syndromes (including unstable angina), coronary angioplasty, or stenting or bypass grafting within six months of Screening.
    - Class III or IV heart failure as defined by the New York Heart Association functional classification system.
    - Uncontrolled hypertension
- Known immediate or delayed hypersensitivity reaction or idiosyncrasy to drugs chemically related to GSK2857916, or any of the components of the study treatment
- Pregnant or lactating female
- Active infection requiring antibiotic, antiviral, or antifungal treatment
- Known HIV infection
- Presence of hepatitis B surface antigen (HBsAg), or hepatitis B core antibody (HBcAb at screening or within 3 months prior to first dose of study treatment
- Positive hepatitis C antibody test result or positive hepatitis C RNA test result at screening or within 3 months prior to first dose of study treatment
    - Note: Participants with positive Hepatitis C antibody due to prior resolved disease can be enrolled, only if a confirmatory negative Hepatitis C RNA test is obtained
    - Note: Hepatitis RNA testing is optional and participants with negative Hepatitis C antibody test are not required to also undergo Hepatitis C RNA testing

BELANTAMAB MAFODOTIN TREATMENT-RELATED CORNEAL EVENTS

In DREAMM-2, a more stringent and objective approach was taken to gain an enhanced understanding of belantamab mafodotin treatment-related corneal events. Corneal event dose modification guidelines were based on a combination of ophthalmic exam findings and changes in visual acuity from baseline (determine by best corrected visual acuity [BCVA] assessment). These objective corneal examination findings (termed here as microcyst-like epithelial changes [MECs]) and BCVA results were combined and graded on the basis of the keratopathy and visual acuity (KVA) scale, When the eye care professional recorded a corneal examination finding, the haematologist/oncologist reported an adverse event (AE) of microcyst-like epithelial keratopathy. Microcyst-like epithelial keratopathy events were mapped to the preferred-term keratopathy per Medical Dictionary for Regulatory Activities (MedDRA). Patients with ophthalmic exam findings such as mild superficial keratopathy or a change of 1 line in visual acuity were permitted to continue treatment at their current dose. However, per protocol, treatment was delayed for patients with ophthalmic exams findings such as moderate/severe punctate keratopathy or peripheral/center sub-epithelial haze or visual acuity changes of 2 or more lines from baseline (not worse than 20/200) until events resolved. Patients were then permitted to reinitiate treatment with a dose reduction. Patient-reported symptoms were also reported per CTCAE scale v4.03,[2] however, these events did not contribute to the dose modification guidelines. Patients received topical corticosteroids prophylaxis (prednisolone acetate 1%, prednisolone phosphate 1%, dexamethasone 0.1%, or equivalent, 1 drop, 4 times daily, starting 1-day predose, for a total of 7 days) and preservative-free lubricant eye drops (1 drop, 4–8 times daily, beginning on Cycle 1 Day 1 until end of treatment) in both eyes. At the start of infusion, cooling eye masks could be applied as a vasoconstrictive measure to reduce possible diffusion of belantamab mafodotin into the cornea.[3]

Table S1. Serious Adverse Events and Adverse Events of Special Interest (Safety Population)[a]

	**Lyophilized belantamab mafodotin 3.4 mg/kg (n=24)**
	Number of patients (%)
**Any Serious Adverse Event (Any Grade)**	15 (63%)
Thrombocytopenia	3 (13%)
Dehydration	2 (8%)
Pneumonia	1 (4%)
Pneumonia influenzal	1 (4%)
Non-cardiac chest pain	1 (4%)
Arthritis	1 (4%)
Haematoma muscle	1 (4%)
Muscular weakness	1 (4%)
Osteonecrosis of jaw	1 (4%)
Large intestinal haemorrhage	1 (4%)
Large intestinal obstruction	1 (4%)
Pancreatitis	1 (4%)
Clavicle fracture	1 (4%)
Subdural haematoma	1 (4%)
Headache	1 (4%)
Spinal cord compression	1 (4%)
Pleural effusion	1 (4%)
Atrial fibrillation	1 (4%)
Cardiac failure	1 (4%)
Haematoma	1 (4%)
Basal cell carcinoma	1 (4%)
Keratopathy (MECs)[a]	1 (4%)
Hypercalcaemia	1 (4%)
Hyperglycaemia	1 (4%)
Hyponatremia	1 (4%)
**Adverse Events of Special Interest (Any Grade)**	
Thrombocytopenia[b]	11 (46%)
Thrombocytopenia	8 (33%)
Platelet count decreased	3 (13%)
Haematoma	1 (4%)
Infusion-related reactions[c]	4 (17%)
Pyrexia	2 (8%)
Infusion-related reaction	2 (8%)
Chills	1 (4%)
Transfusion reaction	1 (4%)
Corneal events	
Keratopathy (MECs)[d]	23 (96%)
Dry eye[e]	6 (25%)
Dry eye	5 (21%)
Eye pruritus	1 (4%)
Blurred vision[f]	9 (38%)
Vision blurred	8 (33%)

Diplopia	2 (8%)
Visual acuity reduced	1 (4%)

MEC, microcyst-like epithelial change.

[a]Events reported based on Common Terminology Criteria for Adverse Events criteria v4.03[2], with the exception of keratopathy (MECs), in the safety population (including all patients who received at least one dose of trial treatment).

[b]Thrombocytopenia included thrombocytopenia, platelet count decreased and haematoma.

[c]Infusion-related reactions included preferred terms infusion-related reaction, pyrexia, chills, and transfusion reaction occurring ≤24 hours of infusion.

[d]Corneal epithelium changes observed on eye examination with or without changes in BCVA from baseline or symptoms. Graded per keratopathy and visual acuity (KVA) scale.

[e]Dry eye included preferred terms dry eye and eye pruritus.

[f]Blurred vision included preferred terms vision blurred, diplopia and visual acuity reduced.

Table S2. List of Sites, Study Investigators and Patients Recruited at Each Site

Country	Institution	Principal investigator	Patients recruited (N=25)
United States	Dana Farber Cancer Institute	RICHARDSON, Paul G	5
United States	MD Anderson Cancer Center, Houston	LEE, Hans C	5
United States	University of Kansas Cancer Center	ABDALLAH, Al-Ola	4
United States	Abramson Cancer Center	COHEN, Adam D	3
United States	Mayo Clinic	KAPOOR, Prashant	3
United States	Levine Cancer Institute, Atrium Health	VOORHEES, Peter M	2
United States	Memorial Sloan Kettering Cancer Center	HULTCRANTZ, Malin[a]	1
Australia	University of Melbourne, St Vincent's Hospital	QUACH, Hang	1
United States	Yale University School of Medicine	NEPARIDZE, Natalia	1

[a]During the study, the principle investigator changed from Nikoletta Lendvai to Malin Hultcrantz.

REFERENCES

1. Rajkumar SV, Dimopoulos MA, Palumbo A, et al. International Myeloma Working Group updated criteria for the diagnosis of multiple myeloma. The Lancet Oncology. 2014;15: e538-e548.
2. Common Terminology Criteria for Adverse Events (CTCAE), Version 4.03. Rockwell, MD: National Cancer Institute. 2009.
3. Zhao H, Atkinson J, Gulesserian S, et al. Modulation of Macropinocytosis-Mediated Internalization Decreases Ocular Toxicity of Antibody–Drug Conjugates. Cancer Research. 2018;78: 2115-2126.
